# Master Regulators Connectivity Map: A Transcription Factors-Centered Approach to Drug Repositioning

**DOI:** 10.3389/fphar.2018.00697

**Published:** 2018-07-02

**Authors:** Marco A. De Bastiani, Bianca Pfaffenseller, Fabio Klamt

**Affiliations:** ^1^Laboratory of Cellular Biochemistry, Department of Biochemistry, Federal University of Rio Grande do Sul, Porto Alegre, Brazil; ^2^National Institute of Science and Technology for Translational Medicine, Porto Alegre, Brazil; ^3^Laboratory of Molecular Psychiatry, Clinicas Hospital of Porto Alegre, Federal University of Rio Grande do Sul, Porto Alegre, Brazil

**Keywords:** connectivity map, computational drug repositioning, master regulators, transcription factors, reverse engineering, systems pharmacology

## Abstract

Drug discovery is a very expensive and time-consuming endeavor. Fortunately, recent omics technologies and Systems Biology approaches introduced interesting new tools to achieve this task, facilitating the repurposing of already known drugs to new therapeutic assignments using gene expression data and bioinformatics. The inherent role of transcription factors in gene expression modulation makes them strong candidates for master regulators of phenotypic transitions. However, transcription factors expression itself usually does not reflect its activity changes due to post-transcriptional modifications and other complications. In this aspect, the use of high-throughput transcriptomic data may be employed to infer transcription factors-targets interactions and assess their activity through co-expression networks, which can be further used to search for drugs capable of reverting the gene expression profile of pathological phenotypes employing the connectivity maps paradigm. Following this idea, we argue that a module-oriented connectivity map approach using transcription factors-centered networks would aid the query for new repositioning candidates. Through a brief case study, we explored this idea in bipolar disorder, retrieving known drugs used in the usual clinical scenario as well as new candidates with potential therapeutic application in this disease. Indeed, the results of the case study indicate just how promising our approach may be to drug repositioning.

## Introduction

Customary approaches to drug development focus on identification of a new treatment target, followed by a search for a compound capable of modulating that target and lastly a validation process. Additional targets for these drugs are not usually investigated, and other clinical applications are not frequently explored. However, these extra elements represent an opportunity for the systematic identification of new indications for existing therapeutics.

The practice of identifying additional therapeutic indications for existing drug compounds, referred to as drug repositioning or repurposing, has some key benefits over traditional methods of drug development (Ashburn and Thor, 2004; Chong and Sullivan, 2007; Jin and Wong, 2014). Indeed, the development process for a repositioned drug can be as short as 3 years, mostly because several steps of the development pipeline can be eliminated during repurposing efforts (Dudley et al., 2011). Additionally, bioinformatics approaches developed in the last 10 years represent powerful, fast and cheap strategies for predicting and choosing new therapeutic indication candidates for existing medications.

Computational approaches may exploit known links between diseases and drugs, which can be used to generalize existing treatments into new clinical contexts. Those diseases–drugs connections can arise by characterizing drugs according to their impact on molecular activity, framing them as perturbations to the biological system. This can identify characteristic signatures for that compound, which can be used to compare many medications, resulting in several opportunities to redirect therapeutic indications between “related” drugs (Readhead and Dudley, 2013). Inserted in this computational approach is an emerging perspective that the understanding of biology and the identification of true drivers of pathologies will require the construction of relevant networks (Schadt and Bjorkegren, 2012).

In this context, transcription factors act as drivers of pathological conditions by modulating overall gene expression. Hence, assembling networks based on co-expression of transcription factors and their target genes may help narrowing down important biological modules unpaired in different diseases (Lopez-Kleine et al., 2013). These gene modules can offer the opportunity for *in silico* screening of drug compounds by simulating the extended effects a given drug may impose on the biological system. In fact, we suggest that gene co-expression networks centered on master regulator transcription factors may be used to identify promising candidates for drug repositioning through a module-oriented adaptation of classical Connectivity Maps. Additionally, we implement a case study of this proposal in the context of bipolar disorder, a complex psychiatric disease, in order to exemplify the potential of this approach for molecules selection.

## Master Regulators of Transcription

Since Susumu Ohno’s first applications of the term “master regulator” or “master regulatory gene” to describe a gene that occupies the very top of a regulatory hierarchy, re-definitions of this concept have emerged to accommodate broader biological facets. One such extended description positions master regulators as participants in the specification of cellular lineages by regulating multiple downstream genes either directly or through a cascade of gene expression changes, ultimately retaining the ability to re-specify the fate of cells (Chan and Kyba, 2013).

Changes in mRNA profiles are a key feature for phenotype characterization from a cell type to another during development, for example. The same rationale may be applied to physiological to pathological transitions in biological systems. In this context, gene expression changes are ultimately mediated and regulated by the activity of transcription factors, which enable a relatively small number of molecules to generate a large diversity of cell types and phenotypic states (Yeh et al., 2013; Bhagwat and Vakoc, 2015; Reiter et al., 2017). Indeed, in several biological systems, such as embryonic stem cells (Muller et al., 2008) or glioblastoma (Carro et al., 2010; Rooj et al., 2016), it was observed that a small number of transcription factors act as master regulators that manage cellular outcome.

In this aspect, previous literature have observed that, given differential gene expression profiles from two independent studies, there was virtually no statistical significance in the overlap between them and these signatures performed poorly in classifying samples from the other study (Michiels et al., 2005; Lim et al., 2009; Padi and Quackenbush, 2015). This observation fits well with the idea of transcription factors acting as master regulators, supporting an approach of exploring the controllers of expression profiles, rather than simply evaluating all differentially expressed genes between two phenotypes of interest. However, the biological activity of transcription factors may not be directly correlated with their expression levels. For that reason, inference of activity is often assessed through expression modifications of the transcription factors’ target genes by reverse engineering methods (Fletcher et al., 2013; Wong et al., 2013; Padi and Quackenbush, 2015; Castro et al., 2016; Senbabaoglu et al., 2016). These approaches can help uncover potentially relevant regulatory units and biological consequences (**Supplementary Figure S1**).

The application of such view in the search for biological markers of phenotypic states has provided new insights in many biomedical investigations, such as cancer (Fletcher et al., 2013; Castro et al., 2016; Chen et al., 2016; Udyavar et al., 2017), diabetes (Piao et al., 2012), and bipolar disorder (Pfaffenseller et al., 2016).

## Systems Pharmacology and Computational Drug Repositioning

The usual “one disease, one target, one drug” paradigm of drug discovery clashes with the novel views of biology, failing to yield effective medications for many complex conditions such as cancer and neurodegenerative diseases (Yildirim et al., 2007). On the other hand, a new archetype of drug research has emerged in recent years, named *Systems Pharmacology*. This paradigm offers an integrated system-level way to drug repurposing or new drugs identification, and facilitates prediction of effectiveness and security of compounds during all phases of development (van der Graaf and Benson, 2011; Zhou et al., 2016). Additionally, it exploits a feature of drugs that for many years has been labeled undesirable: that they often affect more than one molecular target. In fact, this promiscuity, known as polypharmacology, seems to be intrinsic to several drugs’ therapeutic efficacy (Hopkins, 2009).

For drug repositioning, the seminal article of Lamb and collaborators introduced the concept of molecular connectivity map (CMap) (Lamb et al., 2006). The great adherence of the community toward this new idea can be attributed to its embrace of the *Systems Biology* paradigm, which accepts that biological elements have several interdependencies and are effectively connected. In addition, this idea heralds that attempts to defeat such notion by breaking the elements with a single targeted intervention are probably ineffective. Hence, they proposed the need to switch the entire state of the system to a more favorable one, through modulation of many targets simultaneously (Lamb, 2007). Recently, the Library of Integrated Network-based Cellular Signatures (LINCS) project, funded by the National Institutes of Health, expanded the original databases of drug perturbation and enabled the generation of approximately one million gene expression profiles using the L1000 technology^1^ (Ma’ayan et al., 2014; Vempati et al., 2014; Li et al., 2016).

Although the gene expression-based high-throughput approach has the potential to transform biomedicine and accelerate drug discovery (Iorio et al., 2009; Wen et al., 2015; Gillet et al., 2016; Raghavan et al., 2016), the usual workflow relies heavily on signatures of differentially expressed genes and, as mentioned above, differential expression profiles may be prone to poor reproducibility. On the other hand, network-based approaches provide an enriched biological rationale by contextualizing pathologically altered molecular nodes into a systemic functional scenario, possibly enhancing the robustness of drug predictions (Zickenrott et al., 2016). Furthermore, the community has recently been exploring modularity, an important feature of systems network, in the context of connectivity maps. In a network, modules represent highly interconnected local regions (Barabasi et al., 2011), which in the biological context can be easily understood when thinking of pathways. In this aspect, Jadamba and Shin developed a method that identifies disease-specific pathways, by integrating multiple gene expression profiles, and employing them to define pathway-drug networks using semisupervised learning. They tested this proposed pathway-based drug repositioning process in breast cancer and retrieved many known anticancer drugs as well as several new repurposing candidates (Jadamba and Shin, 2016). Chung and collaborators have also devised an interesting approach using gene modules to query the connectivity map, which they named Functional Module Connectivity Map (FMCM). They tested their method against the common practice of selecting drugs using a genomic signature represented by a single set of individual genes and observed that FMCM had higher robustness, accuracy, specificity, and reproducibility in identifying known anti-cancer agents (Chung et al., 2014).

**FIGURE 1:**
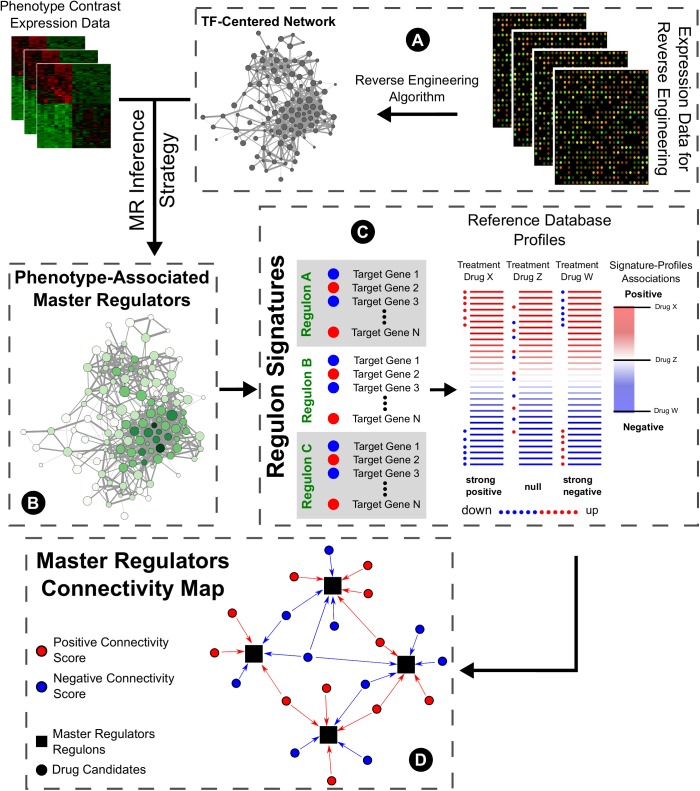
General master regulators connectivity map pipeline. **(A)** Expression data from high-throughput sources are submitted to reverse engineering inference algorithms to predict targets of known transcription factors, constituting regulons. **(B)** Using these regulons, master regulators of pathological phenotypes may be selected by using different strategies (such as GSEA) and data from case-control studies. **(C)** In the CMap original proposal, users query lists of genes whose expression correlates with a biological state of interest and assess their similarity to a reference collection of gene-expression profiles from cultured human cells treated with 1000s of bioactive small molecules. Here, we propose the use of master regulators’ targets expressions to inquire new drug prospects for repurposing. **(D)** The rationale of this connectivity map follows the modulation of the inferred targets of the master regulators transcription factors by the drug candidates.

The concept of transcription factors acting as master regulators of the phenotypic specification overlaps the concept of modularity when reverse engineering methods are used to infer their potential targets. In this context, the inferred targets form a modular unit centered on the transcription factor, comprising a regulon or regulatory unit, under the control of this molecule. Furthermore, if this is a deregulated master regulator of the pathological state, the expression profile of the targets is also altered favoring the disease. Therefore, employing the connectivity maps idea of reversing this profile is an interesting approach to search for potential therapeutic drug repurposing. Thus, the goal of this approach aims for treatments to reverse downstream effectors of disease phenotype by modulating regulatory units of the transcription factors acting as master regulators of the pathology (**Figure 1**).

## Master Regulators Connectivity Map

**FIGURE 2:**
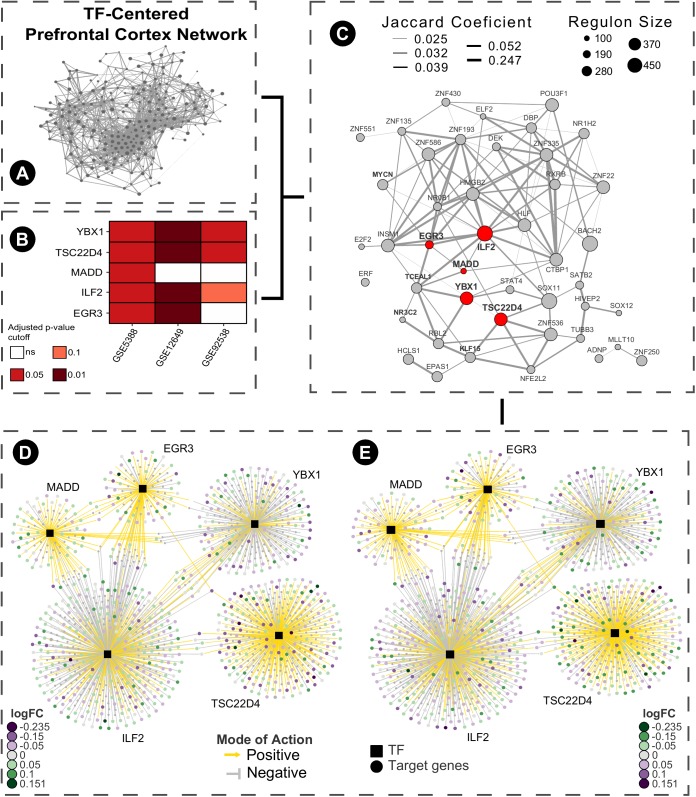
Bipolar disease master regulators connectivity map pipeline. **(A)** Human prefrontal cortex transcriptional network model was computed centered on transcription factors from a large-scale microarray data obtained from Gene Expression Omnibus (GSE30272) using RTN package ARACNe algorithm with 200 permutations and permutation *p*-value < 1e-06 (remaining network reconstruction parameters were kept at default values). **(B)** Regulons of EGR3, ILF2, MADD, TSC22D4, and YBX1 were tested using GSEA in three different datasets of case-control (GSE5388, GSE12649, and GSE92538). **(C)** Samples from these studies were merged based on their common genes, batch corrected using the sva package and a sub-graph of the regulatory units with more than 100 genes was created. **(D)** The inferred TF-target association network of the five selected regulons was extracted and the targets’ logFC direction were inputted as query for the connectivity map using PharmacoGx package using GSEA method and 1000 permutations. **(E)** Following the connectivity map propose, the drugs obtained ideally revert the expression profiles of the pathologically altered regulatory units toward the normal phenotype.

As a brief example of application, we used a Master Regulators Connectivity Map (MRCMap) framework to query potential drugs for repositioning in bipolar disorder. For such, we reproduced the procedures described in Pfaffenseller et al. (2016). Summarily, a tissue-specific transcriptional network model was computed from a large-scale human prefrontal cortex microarray dataset (Colantuoni et al., 2011) using the RTN package available from Bioconductor (Fletcher et al., 2013; Huber et al., 2015; Castro et al., 2016) and afterward we queried the five master regulators regulons previously reported as enriched in bipolar disorder (EGR3, TSC22D4, ILF2, YBX1, and MADD) in two new studies (GSE12649 and GSE92538) besides GSE5388, using Gene Set Enrichment Analysis (GSEA). Considering that usual psychiatric disorder transcriptomic profiles show low to moderate single gene expression changes, this approach enable information extraction and evaluation of data even in such scenarios. In effect, we could observe a satisfactory reproducibility of most regulons in GSE12649, though only two showed significant enrichment (adjusted *p*-value < 0.05) in GSE92538 (Iwamoto et al., 2005; Ryan et al., 2006; Udyavar et al., 2017). Afterward, we merged bipolar and control samples from all three datasets and investigated the connectivity map of these regulatory units. For that, the targets’ logFC direction of all five regulons were assembled and inputted in the R package PharmacoGx (Smirnov et al., 2016). Hence, we aimed for drug candidates that would revert the expression of all five master regulators candidates simultaneously (**Figure 2**). The full list of drugs obtained from the analysis is showed in **Supplementary Table S1**.

Recent meta-analyses of randomized, double-blind studies demonstrated that antipsychotics were significantly more effective than mood stabilizers in the treatment of acute mania, as demonstrated by the superior efficacy profile of risperidone, olanzapine, and haloperidol (Cipriani et al., 2011; Yildiz et al., 2015). Typical antipsychotics block dopamine D2 receptors presenting anti-manic and anti-psychotic effects in acute mania (Tohen and Vieta, 2009), and atypical antipsychotics are antagonists of dopamine D2 receptors as well, but also block type-2 serotonin (5-HT2) receptors (Markowitz et al., 1999). Although their mechanisms of action are still not completely understood, these meta-analyses have supported the recommendation to use dopamine antagonist/partial agonists to treat mania (Goodwin et al., 2016). In this sense, it is not surprising that the MRCMap returned several antipsychotics with potential to modulate regulons enriched in bipolar disorder, including two of the classical and still frequently used typical antipsychotics chlorpromazine and haloperidol (Tohen and Vieta, 2009; Cipriani et al., 2011). Our CMap adaptation also found compounds with antidepressive effects such as maprotiline, mianserin, and desipramine (**Table 1**).

**Table 1:** Master regulators connectivity map results.

Drug	Connectivity score	*p*-Value	^∗^ATC level 1	^∗^ATC level 3	CAS number
Chlorpromazine	-0.270	0.00546	N = NERVOUS SYSTEM	N05A = ANTIPSYCHOTICS	50-53-3
Levomepromazine	-0.258	0.00790	N = NERVOUS SYSTEM	N05A = ANTIPSYCHOTICS	60-99-1
Perphenazine	-0.255	0.01623	N = NERVOUS SYSTEM	N05A = ANTIPSYCHOTICS	58-39-9
Zuclopenthixol	-0.228	0.03376	N = NERVOUS SYSTEM	N05A = ANTIPSYCHOTICS	53772-83-1
Haloperidol	-0.243	0.01810	N = NERVOUS SYSTEM	N05A = ANTIPSYCHOTICS	52-86-8
Promazine	-0.236	0.02966	N = NERVOUS SYSTEM	N05A = ANTIPSYCHOTICS	58-40-2

Maprotiline	-0.253	0.00566	N = NERVOUS SYSTEM	N06A = ANTIDEPRESSANTS	10262-69-8
Desipramine	-0.226	0.02894	N = NERVOUS SYSTEM	N06A = ANTIDEPRESSANTS	50-47-5
Mianserin	-0.232	0.01657	N = NERVOUS SYSTEM	N06A = ANTIDEPRESSANTS	24219-97-4

Diflorasone	-0.207	0.03009	D = DERMATOLOGICALS	D07A = CORTICOSTEROIDS, PLAIN	2557-49-5
Meclofenamic acid	-0.236	0.02462	M = MUSCULO-SKELETAL SYSTEM	M01A = ANTIINFLAMMATORY AND ANTIRHEUMATIC PRODUCTS, NON-STEROIDS	644-62-2
Ketorolac	-0.222	0.04700	M = MUSCULO-SKELETAL SYSTEM	M01A = ANTIINFLAMMATORY AND ANTIRHEUMATIC PRODUCTS, NON-STEROIDS	74103-06-3
Trolox c	-0.248	0.00341			53188-07-1
Acetylsalicylsalicylic acid	-0.246	0.00472			530-75-6

*Drugs with antimaniac effects. Drugs with antidepressive effects. Drugs with anti-inflammatory and/or antioxidant effects. ^∗^Anatomical therapeutic chemical (ATC) classification.*

Despite the availability of several effective drugs for the management of acute mania, most pharmacological drugs currently used to treat psychiatric disorders act through mechanisms discovered a long time ago, usually acting at neurotransmitter receptors that may modulate several signal transduction pathways and induce different responses (Geddes and Miklowitz, 2013). Nevertheless, molecules targeting specific signal transduction pathways, not necessarily related to known traditional mechanisms of psychiatric drugs, may be interesting therapeutic approaches. We have also identified some drugs that act on pathways possibly involved in bipolar disorder pathophysiology, such as inflammatory and oxidative stress pathways (Berk et al., 2011). These include: non-steroidal anti-inflammatory agents (meclofenamic acid, ketorolac and acetylsalicylsalicylic acid, a degradation product of aspirin), a steroid anti-inflammatory agent with anti-inflammatory and immunosuppressive properties (diflorasone) and a molecule with antioxidant profile (trolox C).

Immune disturbances have been strongly suggested as an important component for the high prevalence of medical comorbidities in bipolar disorder and for its pathophysiology (Leboyer et al., 2012; Rosenblat and McIntyre, 2015). In fact, several reports in literature suggest that bipolar disorder is associated with a chronic low-grade inflammation (Brietzke et al., 2009; Modabbernia et al., 2013; Munkholm et al., 2013; Barbosa et al., 2014). Furthermore, studies have shown antidepressant effects of adjunctive agents with anti-inflammatory properties in bipolar disorder (Keck et al., 2006; Berk et al., 2008; Nery et al., 2008; Savitz et al., 2012). Current pharmacologic therapy for bipolar disorder involves low tolerability and high rates of treatment resistance with recurrent depressive episodes (Gitlin, 2006). Thus, novel and interesting targets for a better management of bipolar disorder may involve molecules that act on the inflammatory pathways, such as those identified in the MRCMap analysis.

## Concluding Remarks

Prompted by the prohibitive costs and time consuming pitfalls of traditional approaches, recent years have unraveled new ways to tackle the drug discovery and development issue, centered on information integration and analysis, and leading to computational repositioning strategies. This novel paradigm shows great multidisciplinary characteristics, incorporating several current hot topics on biology, statistics, applied mathematics, and informatics. In this context, data generated by high-throughput technologies and computational methods to integrate and analyze them have played an important role. Moreover, the current systems view of biology promises more holistic, efficient, and rational avenues of research.

Following this idea, we propose the use of transcription factors acting as master regulators of pathological states as proxies to query new drugs for repurposing. The regulatory units of these master regulators, inferred through reverse engineering, may be explored with current connectivity maps approaches as a biologically functional groups of genes, which pathological expressions we would like to revert. Of additional importance is the possibility to integrate several layers of biological complexity (Padi and Quackenbush, 2015) to improve and refine the primary workflow showed in **Figure 1**. Since the outcome relies on the reconstruction of regulatory TF-target associations, incorporating strategies to enhance the resolution of these interactions using protein-binding microarray (Wong et al., 2013) and/or proteomics are a very interesting prospect to develop this type of modeling. Also, even though our case study retrieved several drugs currently used in BD with only the expression data and the regulatory network reconstruction, we believe the pipeline proposed could be further improved by adding different network analyses. **Table 2** qualifies a few advantages and disadvantages of adopting such strategy.

**Table 2:** Advantages and disadvantages of TF-centered CMap versus standard differentially expressed gene signature CMap.

Advantages	Disadvantages
Enables sophisticated modeling strategies through reconstruction of gene regulatory networks.	Requires more sophisticated bioinformatics analyses prior to CMap phase.
Enables the incorporation of network biology complexity to drug discovery.	Requires extended computation pipelines and expertise.
By incorporating transcription factors rationale as master regulators of groups of genes, enables extended biologically relevant knowledge to accompany the drug selection process.	Requires careful parameterization during regulatory network reconstruction phase.
Enables extensive integration of external data from many other types and sources (e.g., protein-binding microarray, proteomics, and epigenetics) to improve selection robustness and validity.	

Using bipolar disorder as a short case study, we have retrieved several drugs with potential to revert regulatory units previously proposed as master regulators of this disease, among which were antipsychotics, antidepressive, anti-inflammatory, and anti-oxidant agents. Some of these molecules are current clinical therapies for bipolar disorder (e.g., haloperidol and chlorpromazine), while other present new opportunities of investigation. It is important to note that standard differentially expressed CMap of the top 500 genes in the merged BD dataset queried using build2^2^ did not retrieve drugs such as haloperidol and chlorpromazine (**Supplementary Table S2**). However, more studies are required to further consolidate the proposed framework and fully assess the validity of the new repositioning candidates retrieved in experimental/clinical scenarios.

Finally, although the regulatory units of master regulators present an interesting new approach to evaluate repurposing of drugs using connectivity maps, some caution remarks are required when employing this strategy. Since reverse engineering of regulatory networks is a new and growing field of systems biology research, the algorithms used to infer of the master regulators’ regulatory units during the initial stages of the process may affect the inputs to the connectivity map stage. Thus, careful inspection of the computational parameters and procedures are important to assure reproducibility. Furthermore, CMap also comes with some pitfalls, such as limited drug perturbation data, a limited drug coverage and dosage-dependent conditions, although LINCS project have helped mitigate these factors. Also, besides the uncertainty of employing cell lines expression patterns, usually there is no account for dynamics associated with the disease or the drug under investigation, multi-organ effects, and genetic variations (Musa et al., 2018). Nevertheless, search for repositioning drugs using functional modules centered on transcription factors promises an exciting, rational and biologically relevant strategy, especially as the reverse engineering manual methods advance toward novel, more reliable and powerful computational and statistical stages.

## Data Availability

Datasets used in this study can be accessed via NCBI GEO portal (https://www.ncbi.nlm.nih.gov/geo/). Further intermediate data and codes generated to implement the MRCMap adaptation are available from the corresponding author on request.

## Author Contributions

MDB conceived the CMap adaptation and implementation. BP analyzed and discussed the bipolar disorder case study results. FK reviewed and supervised the analyses. All authors reviewed the final manuscript.

## Conflict of Interest Statement

MDB’s Ph.D. student scholarship was supported by Conselho Nacional de Pesquisa (CNPq); this work was also supported by INCT-TM CNPq/FAPESP (#465458/2014-9) and Pesquisas Sobre Doenças Neurodegenerativas (#466989/2014-8). The remaining authors declare that the research was conducted in the absence of any commercial or financial relationships that could be construed as a potential conflict of interest.

## Footnotes

1 http://lincsproject.org/

2 https://portals.broadinstitute.org/cmap/

## Supplementary Material

FIGURE S1 TF-Centered Reverse Engineered Network. Expression data obtained from high-throughput technologies may be used to build transcription factors-centered networks. **(A)** First, expression information of known genes with transcription factor activity and their potential targets are divided and **(B)** TF-target association status can be computed employing reverse engineering methodologies such as Bayesian networks, mutual information, or others (Senbabaoglu et al., 2016). Different network representations of these computations can summarize several aspects of biological complexity. Regulon association network **(C)** shows how regulatory units connect to each other through common targets (nodes represent the number of targets inferred for that TF and edges are associated with the number of overlapped targets between any two TF). Transcription factors regulatory network **(D)** representation shows the type of association inferred between transcription factors and its targets in a regulatory unit.

TABLE S1 Master regulators connectivity map results.

TABLE S2 Standard CMap of top 500 differentially expressed genes in merged Bipolar Disorder dataset.
