# Supplementary figures and images for: Master Regulators Connectivity Map: A Transcription Factors-Centered Approach to Drug Repositioning
Source: Front Pharmacol. 2018 Jul 2;9:697. doi: 10.3389/fphar.2018.00697 (PMC6043797; doi:10.3389/fphar.2018.00697)

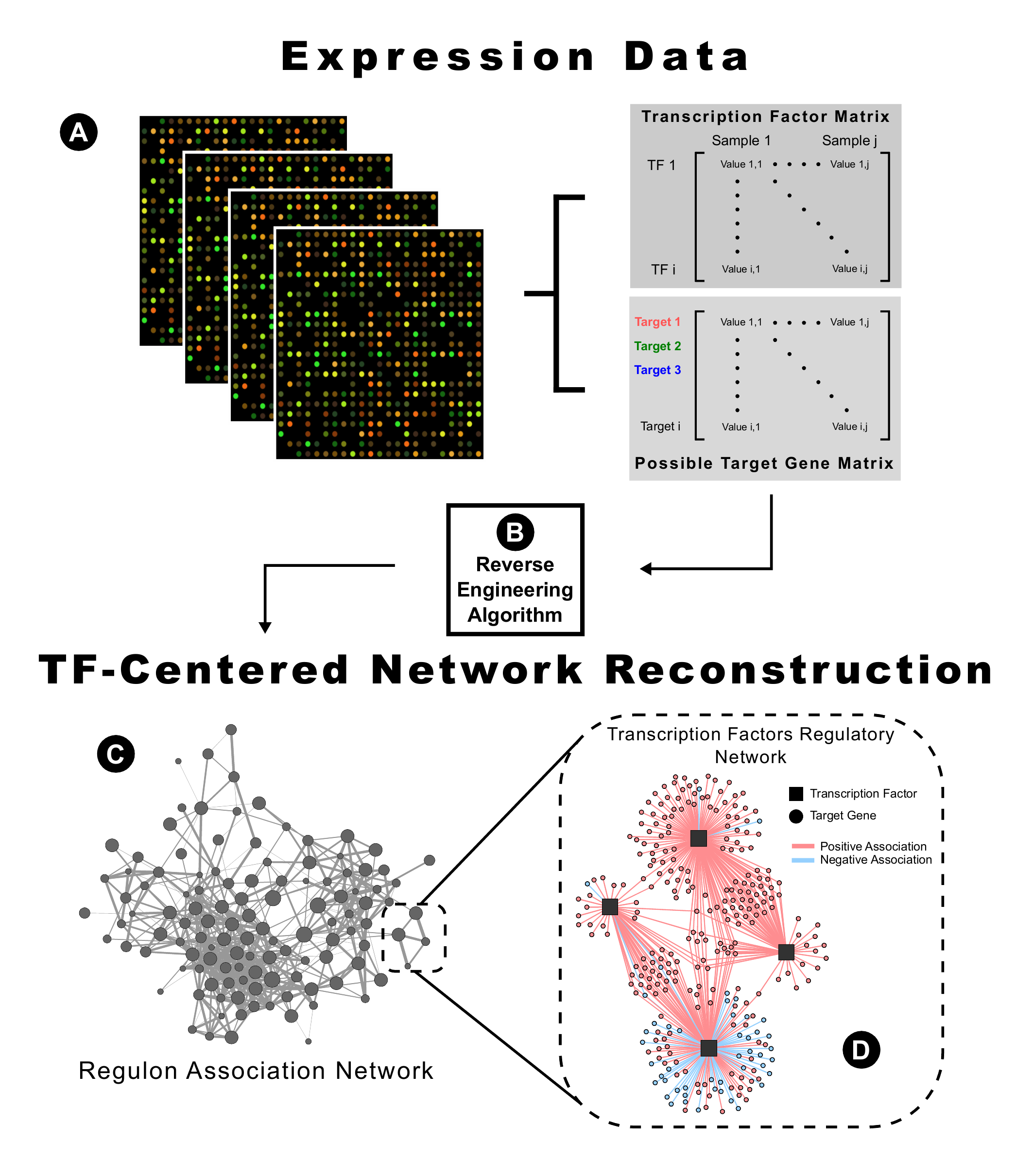

Supplement: FIGURE S1 — TF-Centered Reverse Engineered Network. Expression data obtained from high-throughput technologies may be used to build transcription factors-centered networks. (A) First, expression information of known genes with transcription factor activity and their potential targets are divided and (B) TF-target association status can be computed employing reverse engineering methodologies such as Bayesian networks, mutual information, or others (Senbabaoglu et al., 2016). Different network representations of these computations can summarize several aspects of biological complexity. Regulon association network (C) shows how regulatory units connect to each other through common targets (nodes represent the number of targets inferred for that TF and edges are associated with the number of overlapped targets between any two TF). Transcription factors regulatory network (D) representation shows the type of association inferred between transcription factors and its targets in a regulatory unit. [file Image_1.TIF]
