# Supplementary material for: Master Regulators Connectivity Map: A Transcription Factors-Centered Approach to Drug Repositioning
Source: Front Pharmacol. 2018 Jul 2;9:697. doi: 10.3389/fphar.2018.00697 (PMC6043797; doi:10.3389/fphar.2018.00697)
Supplement: TABLE S1 — Master regulators connectivity map results. [file Table_1.DOCX]

| **Supplementary Table 1. Master Regulators Connectivity Map Results (FULL TABLE)** | | | | | | |
| --- | --- | --- | --- | --- | --- | --- |
| Drug | Connectivity Score | p-value | ^*^ATC level 1 | ^*^ATC level 2 | ^*^ATC level 3 | CAS number |
| loperamide | -0.248 | 0.01971 | A=ALIMENTARY TRACT AND METABOLISM | A07=ANTIDIARRHEALS, INTESTINAL ANTIINFLAMMATORY/ANTIINFECTIVE AGENTS | A07D=ANTIPROPULSIVES | 53179-11-6 |
| dicoumarol | -0.244 | 0.00554 | B=BLOOD AND BLOOD FORMING ORGANS | B01=ANTITHROMBOTIC AGENTS | B01A=ANTITHROMBOTIC AGENTS | 66-76-2 |
| xamoterol | -0.230 | 0.03101 | C=CARDIOVASCULAR SYSTEM | C01=CARDIAC THERAPY | C01C=CARDIAC STIMULANTS EXCL. CARDIAC GLYCOSIDES | 81801-12-9 |
| furosemide | -0.241 | 0.00167 | C=CARDIOVASCULAR SYSTEM | C03=DIURETICS | C03C=HIGH-CEILING DIURETICS | 54-31-9 |
| bumetanide | -0.238 | 0.00482 | C=CARDIOVASCULAR SYSTEM | C03=DIURETICS | C03C=HIGH-CEILING DIURETICS | 28395-03-1 |
| naftidrofuryl | -0.251 | 0.00284 | C=CARDIOVASCULAR SYSTEM | C04=PERIPHERAL VASODILATORS | C04A=PERIPHERAL VASODILATORS | 31329-57-4 |
| lidoflazine | -0.251 | 0.00165 | C=CARDIOVASCULAR SYSTEM | C08=CALCIUM CHANNEL BLOCKERS | C08E=NON-SELECTIVE CALCIUM CHANNEL BLOCKERS | 3416-26-0 |
| lovastatin | -0.233 | 0.02459 | C=CARDIOVASCULAR SYSTEM | C10=LIPID MODIFYING AGENTS | C10A=LIPID MODIFYING AGENTS, PLAIN | 75330-75-5 |
| econazole | -0.237 | 0.01629 | D=DERMATOLOGICALS | D01=ANTIFUNGALS FOR DERMATOLOGICAL USE | D01A=ANTIFUNGALS FOR TOPICAL USE | 27220-47-9 |
| clemastine | -0.238 | 0.03361 | D=DERMATOLOGICALS | D04=ANTIPRURITICS, INCL. ANTIHISTAMINES, ANESTHETICS, ETC. | D04A=ANTIPRURITICS, INCL. ANTIHISTAMINES, ANESTHETICS, ETC. | 15686-51-8 |
| fusidic acid | -0.223 | 0.02937 | D=DERMATOLOGICALS | D06=ANTIBIOTICS AND CHEMOTHERAPEUTICS FOR DERMATOLOGICAL USE | D06A=ANTIBIOTICS FOR TOPICAL USE | 6990-06-3 |
| diflorasone | -0.207 | 0.03009 | D=DERMATOLOGICALS | D07=CORTICOSTEROIDS, DERMATOLOGICAL PREPARATIONS | D07A=CORTICOSTEROIDS, PLAIN | 2557-49-5 |
| fluticasone | -0.229 | 0.04845 | D=DERMATOLOGICALS | D07=CORTICOSTEROIDS, DERMATOLOGICAL PREPARATIONS | D07A=CORTICOSTEROIDS, PLAIN | 90566-53-3 |
| benzethonium chloride | -0.278 | 0.00576 | D=DERMATOLOGICALS | D08=ANTISEPTICS AND DISINFECTANTS | D08A=ANTISEPTICS AND DISINFECTANTS | 121-54-0 |
| tretinoin | -0.264 | 0.00400 | D=DERMATOLOGICALS | D10=ANTI-ACNE PREPARATIONS | D10A=ANTI-ACNE PREPARATIONS FOR TOPICAL USE | 302-79-4 |
| ivermectin | -0.264 | 0.00198 | D=DERMATOLOGICALS | D11=OTHER DERMATOLOGICAL PREPARATIONS | D11A=OTHER DERMATOLOGICAL PREPARATIONS | 70288-86-7 |
| benzathine benzylpenicillin | -0.226 | 0.03850 | J=ANTIINFECTIVES FOR SYSTEMIC USE | J01=ANTIBACTERIALS FOR SYSTEMIC USE | J01C=BETA-LACTAM ANTIBACTERIALS, PENICILLINS | 1538-09-6 |
| ceftazidime | -0.216 | 0.03958 | J=ANTIINFECTIVES FOR SYSTEMIC USE | J01=ANTIBACTERIALS FOR SYSTEMIC USE | J01D=OTHER BETA-LACTAM ANTIBACTERIALS | 72558-82-8 |
| lincomycin | -0.241 | 0.00600 | J=ANTIINFECTIVES FOR SYSTEMIC USE | J01=ANTIBACTERIALS FOR SYSTEMIC USE | J01F=MACROLIDES, LINCOSAMIDES AND STREPTOGRAMINS | 154-21-2 |
| clofazimine | -0.242 | 0.01255 | J=ANTIINFECTIVES FOR SYSTEMIC USE | J04=ANTIMYCOBACTERIALS | J04B=DRUGS FOR TREATMENT OF LEPRA | 2030-63-9 |
| Rapamycin | -0.297 | 0.00979 | L=ANTINEOPLASTIC AND IMMUNOMODULATING AGENTS | L04=IMMUNOSUPPRESSANTS | L04A=IMMUNOSUPPRESSANTS | 53123-88-9 |
| meclofenamic acid | -0.236 | 0.02462 | M=MUSCULO-SKELETAL SYSTEM | M01=ANTIINFLAMMATORY AND ANTIRHEUMATIC PRODUCTS | M01A=ANTIINFLAMMATORY AND ANTIRHEUMATIC PRODUCTS, NON-STEROIDS | 644-62-2 |
| acemetacin | -0.219 | 0.02563 | M=MUSCULO-SKELETAL SYSTEM | M01=ANTIINFLAMMATORY AND ANTIRHEUMATIC PRODUCTS | M01A=ANTIINFLAMMATORY AND ANTIRHEUMATIC PRODUCTS, NON-STEROIDS | 53164-05-9 |
| ketorolac | -0.222 | 0.04700 | M=MUSCULO-SKELETAL SYSTEM | M01=ANTIINFLAMMATORY AND ANTIRHEUMATIC PRODUCTS | M01A=ANTIINFLAMMATORY AND ANTIRHEUMATIC PRODUCTS, NON-STEROIDS | 74103-06-3 |
| dyclonine | -0.243 | 0.01433 | N=NERVOUS SYSTEM | N01=ANESTHETICS | N01B=ANESTHETICS, LOCAL | 586-60-7 |
| metixene | -0.285 | 0.00015 | N=NERVOUS SYSTEM | N04=ANTI-PARKINSON DRUGS | N04A=ANTICHOLINERGIC AGENTS | 4969-02-2 |
| trihexyphenidyl | -0.240 | 0.01929 | N=NERVOUS SYSTEM | N04=ANTI-PARKINSON DRUGS | N04A=ANTICHOLINERGIC AGENTS | 144-11-6 |
| prochlorperazine | -0.301 | 0.00173 | N=NERVOUS SYSTEM | N05=PSYCHOLEPTICS | N05A=ANTIPSYCHOTICS | 58-38-8 |
| chlorpromazine | -0.270 | 0.00546 | N=NERVOUS SYSTEM | N05=PSYCHOLEPTICS | N05A=ANTIPSYCHOTICS | 50-53-3 |
| levomepromazine | -0.258 | 0.00790 | N=NERVOUS SYSTEM | N05=PSYCHOLEPTICS | N05A=ANTIPSYCHOTICS | 60-99-1 |
| perphenazine | -0.255 | 0.01623 | N=NERVOUS SYSTEM | N05=PSYCHOLEPTICS | N05A=ANTIPSYCHOTICS | 58-39-9 |
| haloperidol | -0.243 | 0.01810 | N=NERVOUS SYSTEM | N05=PSYCHOLEPTICS | N05A=ANTIPSYCHOTICS | 52-86-8 |
| promazine | -0.236 | 0.02966 | N=NERVOUS SYSTEM | N05=PSYCHOLEPTICS | N05A=ANTIPSYCHOTICS | 58-40-2 |
| zuclopenthixol | -0.228 | 0.03376 | N=NERVOUS SYSTEM | N05=PSYCHOLEPTICS | N05A=ANTIPSYCHOTICS | 53772-83-1 |
| mesoridazine | -0.222 | 0.03528 | N=NERVOUS SYSTEM | N05=PSYCHOLEPTICS | N05A=ANTIPSYCHOTICS | 5588-33-0 |
| thioproperazine | -0.209 | 0.04228 | N=NERVOUS SYSTEM | N05=PSYCHOLEPTICS | N05A=ANTIPSYCHOTICS | 316-81-4 |
| maprotiline | -0.253 | 0.00566 | N=NERVOUS SYSTEM | N06=PSYCHOANALEPTICS | N06A=ANTIDEPRESSANTS | 10262-69-8 |
| mianserin | -0.232 | 0.01657 | N=NERVOUS SYSTEM | N06=PSYCHOANALEPTICS | N06A=ANTIDEPRESSANTS | 24219-97-4 |
| desipramine | -0.226 | 0.02894 | N=NERVOUS SYSTEM | N06=PSYCHOANALEPTICS | N06A=ANTIDEPRESSANTS | 50-47-5 |
| nortriptyline | -0.240 | 0.04380 | N=NERVOUS SYSTEM | N06=PSYCHOANALEPTICS | N06A=ANTIDEPRESSANTS | 72-69-5 |
| tacrine | -0.221 | 0.02861 | N=NERVOUS SYSTEM | N06=PSYCHOANALEPTICS | N06D=ANTI-DEMENTIA DRUGS | 321-64-2 |
| proguanil | -0.233 | 0.03071 | P=ANTIPARASITIC PRODUCTS, INSECTICIDES AND REPELLENTS | P01=ANTIPROTOZOALS | P01B=ANTIMALARIALS | 500-92-5 |
| astemizole | -0.254 | 0.02506 | R=RESPIRATORY SYSTEM | R06=ANTIHISTAMINES FOR SYSTEMIC USE | R06A=ANTIHISTAMINES FOR SYSTEMIC USE | 68844-77-9 |
| iodixanol | -0.235 | 0.00757 | V=VARIOUS | V08=CONTRAST MEDIA | V08A=X-RAY CONTRAST MEDIA, IODINATED | 92339-11-2 |
| Trolox C | -0.248 | 0.00341 |  |  |  | 53188-07-1 |
| ursolic acid | -0.241 | 0.00348 |  |  |  |  |
| 0297417-0002B | -0.364 | 0.00357 |  |  |  | 127967-03-7 |
| piperacetazine | -0.256 | 0.00391 |  |  |  | 3819-00-9 |
| chlorcyclizine | -0.243 | 0.00408 |  |  |  | 82-93-9 |
| acetylsalicylsalicylic acid | -0.246 | 0.00472 |  |  |  | 530-75-6 |
| homochlorcyclizine | -0.257 | 0.00489 |  |  |  | 848-53-3 |
| eticlopride | -0.240 | 0.00713 |  |  |  | 97612-24-3 |
| sulfamonomethoxine | -0.230 | 0.01424 |  |  |  | 1220-83-3 |
| alpha-yohimbine | -0.232 | 0.01666 |  |  |  | 131-03-3 |
| lasalocid | -0.238 | 0.02109 |  |  |  | 25999-31-9 |
| gabexate | -0.209 | 0.02571 |  |  |  | 39492-01-8 |
| spiradoline | -0.229 | 0.02751 |  |  |  | 87151-85-7 |
| thioperamide | -0.207 | 0.03313 |  |  |  | 106243-16-7 |
| Prestwick-1082 ((-)-Eseroline fumarate salt) | -0.220 | 0.03367 |  |  |  | 70310-73-5 |
| metitepine | -0.226 | 0.04174 |  |  |  | 20229-30-5 |
| coralyne | -0.226 | 0.04943 |  |  |  | 6872-73-7 |
| ^*^Anatomical Therapeutic Chemical (ATC) Classification | | | | | | |
